# Association of Major Depressive Disorder on Heart Failure With Reduced and Preserved Ejection Fraction: Analysis of National Readmission Database 2018

**DOI:** 10.7759/cureus.15107

**Published:** 2021-05-19

**Authors:** Harshith S Thyagaturu, Sittinun Thangjui, Kashyap Shah, Riddhima V Naik, Gayatri Bondi

**Affiliations:** 1 Internal Medicine, Bassett Healthcare Network, Cooperstown, USA; 2 Internal Medicine, Saint Luke's University Hospital, Bethlehem, USA

**Keywords:** heart failure, cardiac failure, depression, major depressive disorder, readmission, mortality

## Abstract

Introduction

The effect of major depressive disorder (MDD) on heart failure types is unclear. We aimed to assess the association of depression in heart failure with preserved ejection fraction (HFpEF) and heart failure with reduced ejection fraction (HFrEF) readmissions using the Nationwide Readmission Database (NRD) 2018.

Methods

We identified hospitalizations with a primary discharge diagnosis of HFrEF and HFpEF by appropriate ICD-10-CM codes. We acquired mortality and readmission data with and without MDD at 30 days. We used multivariate logistic regression analysis to estimate the adjusted odds ratio (aOR).

Results

Among 102,997 patients admitted with heart failure as a primary diagnosis, 11% had MDD. We found a similar prevalence of HFpEF with MDD compared to HFrEF at 13.9% and 10%, respectively. Both HFrEF and HFpEF patients with MDD had similar combined outcomes of 30-day mortality and rehospitalization compared to patients without MDD with aOR 0.94 (95% CI: 0.85-1.04) and 0.93 (95% CI: 0.81-1.07), respectively. Both types of HF with MDD were associated with lesser mortality.

Conclusion

MDD was associated with similar combined 30-day mortality and readmissions for both HFrEF and HFpEF. However, MDD was associated with decreased 30-day mortality in both groups of heart failure (HF) patients. Further studies with robust medications and treatment data are needed to verify the results of our study.

## Introduction

Depression has shown to be associated with worsening outcomes in heart failure (HF) patients since early 2000. A meta-analysis in 2006 showed a 22% prevalence of depression in HF patients. The prevalence of depression in this meta-analysis was higher in studies that used the questionnaires (e.g., Beck Depression Scale, Geriatric Depression Scale, etc.) to diagnose depression compared to clinician diagnosis. The depressive disorder was also associated with a greater than a two-fold risk of mortality and associated clinical events [1]. This result was similar to the prevalence studies done during 2007 to 2010 in the United States that showed nearly a two-fold higher risk of hospital admission and emergency department visits and a four-fold increase in mortality in HF patients with moderate to severe depression [2]. However, data from 2010 to 2014 from the Nationwide Readmission Database, which included approximately 60% of hospitalized patients in the United States, showed only a modest increase in readmission rates in patients with depression after adjusting for other comorbidities [3]. Similarly, a Danish database from 1995 to 2014 showed a modest increase in mortality in heart failure patients with depression [4]. Given this significant risk of poor outcome in HF patients with depression, psychosocial intervention and pharmacological treatment were studied for depression in HF patients. Major antidepressant trials using sertraline in heart failure with reduced ejection fraction (HFrEF) and heart failure with preserved ejection fraction (HFpEF) and escitalopram in HF reduced ejection fraction (HFrEF) were performed but, results suggested that it did not significantly reduce depression, mortality or rehospitalization [5,6].

Cognitive-behavioral therapy reduced symptoms of depression and improved HF-related quality of life, but no data on mortality and readmission was noted [7]. In 2016, the European Society of Cardiology guidelines for diagnosing and treating acute and chronic heart failure included depression as significant comorbidity contributing to the development of HF, especially in the elderly [8]. Since then, depression had become well-established comorbidity of HF. However, HFrEF and HFpEF have different etiological profiles and prognoses. In addition, guideline-directed medical therapy (GDMT) with ACEI, ARB, or beta-blockers has proven beneficial in HFrEF, but not HFpEF [9]. Compared with HFrEF, patients with HFpEF are older, more often, women, and more commonly have a history of hypertension and AF [10].

The effect of depression on rehospitalization and mortality between HFrEF and HFpEF is limited to date. We aim to determine the outcome of readmission and mortality in HF patients with and without depression in the current era. We also aimed to identify the prevalence of depression in HF patients using the Nationwide Readmission Database 2018.

## Materials and methods

We conducted a retrospective cohort study using the National Readmission Database (NRD) for the year 2018. The NRD is a database developed for the Healthcare Cost and Utilization Project (HCUP) sponsored by the Agency for Healthcare Research and Quality through a Federal-State-Industry partnership. In 2018, the NRD contained data from 28 geographically dispersed states, accounting for ~60% of the total US resident population and 58.7% of all US hospitalizations [11]. It contains reliable, verifiable patient linkage numbers that could track a patient across hospitals within a state while adhering to strict privacy guidelines. The NRD comprises more than 100 clinical and non-clinical variables for each hospital stay. Each discharge is weighted (weight = total number of discharges from all acute care hospitals in the United States divided by the number of discharges included in the 20% sample) to calculate the national estimates. NRD has been previously used to provide reliable national readmission estimates in heart failure [12,13]. The NRD 2018 contains patient and hospital-level data with up to 40 diagnoses and 25 procedures collected for each patient using the International Classification of Diseases, Tenth Revision, Clinical Modification (ICD10-CM). We did not obtain institutional review board (IRB) approval due to the data’s de-identified nature.

Study population

**Figure 1:**
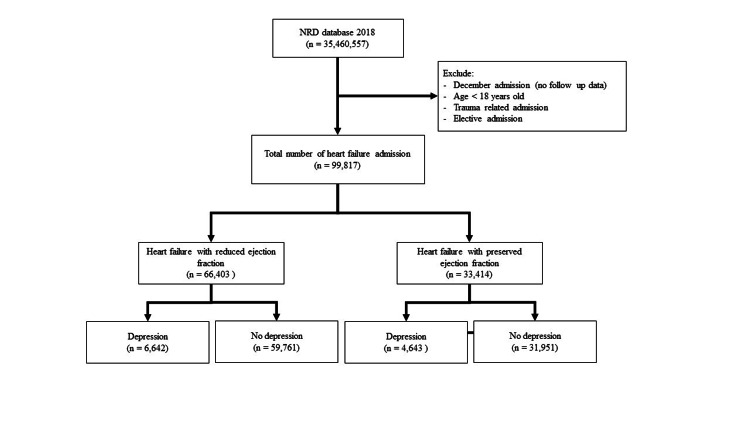
Inclusion diagram NRD = Nationwide Readmissions Database

We included all patients hospitalized with a primary diagnosis of heart failure. We used appropriate ICD-10 CM codes to identify HFrEF and HFpEF as utilized in the previous study (Table 1) [14]. We identified patients with major depressive disorder (MDD) in the secondary diagnosis field. Patients were excluded if they were <18 years old or if the admission was elective. We also excluded patients admitted in December because the NRD captured admission based on a calendar year (i.e., January 1 through December 31) without a link to the previous or following year. We also excluded the readmissions caused by traumatic causes (Figure 1).

**Table 1:** ICD-10 codes used in this study ICD-10 = International Classification of Diseases, Tenth Revision

Variables	ICD-10 codes
Heart failure with a reduced ejection fraction	I501, I502, I504, I5082
Heart failure with a preserved ejection fraction	I503
Major depressive disorder (MDD)	F32, F33
Atrial fibrillation	I480, I4811, I4819, I4891, I482
Coronary artery disease	I20, I21, I22, I23, I24, I25
Essential hypertension	I10, I11, I12, I13, I14, I15, I16
Diabetes mellitus	E08, E09, E10, E11, E13
Chronic obstructive pulmonary disease	J41, J42, J43, J44
Obesity	E66, Z683, Z684
Obstructive sleep apnea	G4733
Chronic kidney disease ≥ Stage III	N183, N184, N185, E082, E132, I12, I13
End-stage renal disease on hemodialysis	N186, Z992, Z4931, Z4901
Hypothyroidism	E03
Alcohol-related disorders	F10
Anemia	D50, D51, D52, D53, D55, D56, D57, D58, D59, D60, D61, D62, D63, D64, D460, D461, D462, D464, O990
History of stroke	I693, Z8673
Peripheral artery disease	E085, E095, E105, E115, E135, I73, T82856, Z9862, Z95820, I252, I2583
Nicotine dependence	F17, Z87891
Cocaine abuse	F14, R782
Schizophrenia	F20
Bipolar disorder	F31
Generalized anxiety disorder	F41
Post-traumatic stress disorder	F431, F432
Attention deficit hyperactivity disorder	F90

Study outcomes

The primary outcome was the composite outcome of all-cause readmission and mortality rate at 30 days for HFrEF with MDD compared to HFrEF without MDD, and then the composite outcome of all-cause readmission and mortality rate at 30 days for HFpEF with MDD compared to HFpEF without MDD. The secondary outcomes were to (a) compare all-cause readmission rate at 30 days for HFrEF and HFpEF with MDD and without MDD; (b) compare all-cause mortality rate at 30 days for HFrEF and HFpEF with MDD and without MDD; (c) compare resource utilization including total hospitalization charges, cost, and length of stay in HFrEF and HFpEF with MDD and without MDD.

Definition of variables

We used variables available in the NRD to identify baseline characteristics, including age, gender, median household income for patient’s zip code, primary expected payer, admission type, admission day of the week, discharge status, and hospital characteristics (such as bed size and teaching status). The patient comorbidities were identified using the ICD10-CM codes (Table 1). The comorbidity burden was assessed using the Elixhauser Comorbidity Index. Readmission was defined as a non-traumatic admission with any principal diagnosis within 30 days of the index admission. If patients had multiple readmissions within 30 days of discharge, only the first readmission was counted. We used the patient’s vital status at discharge for the in-hospital mortality rate, which was directly coded in the database. The three most common reasons for readmission were determined by tallying the principal diagnosis for each readmitted patient. Total hospitalization charges represent the amount that the hospital billed for the entire hospital stay.

Statistical analysis

The NRD is based on an intricate survey design that includes stratification, clustering, and weighting. The variable “discwt” was used to generate national estimates. Standard error calculations were made considering stratification (“nrd_stratum”) and hospitals defining the clusters (“hosp_nrd”). Categorical data were presented as frequency (%) and were compared using the chi-square test. Continuous data were presented in mean ± standard deviation (SD) and were compared using the Student’s t-test. Unadjusted odds ratio (OR) was calculated by univariate logistic regression for primary and secondary outcomes. Multivariate logistic regression was used to calculate the adjusted OR. Variables that were deemed important determinants of the outcomes based on literature were included in the models. In addition, multiple covariates were built into the model if p < 0.2 in univariate analysis. A two-tailed p-value of 0.05 was designated as statistically significant. We adhered to the methodological standard of the Healthcare Cost and Utilization Project (HCUP) [15] and followed the checklist provided by the HCUP [16]. Stata SE, version 16.1 (College Station, Texas: StataCorp.) was used for all statistical analyses.

## Results

From a total of 35,460,557 hospitalizations, 99,817 index admissions of HF were included. Of which, 66,403 admissions were due to HFrEF and 36,594 due to HFpEF. The prevalence of MDD in HFrEF patients was 10%. The prevalence of MDD in HFpEF patients was 13.9%.

Baseline characteristics

Complete data of baseline characteristics of HFrEF and HFpEF cohort are presented in Table 2. Most of the HFrEF cohort patients were older than 75 years in the MDD and non-MDD groups. The mean age of HFrEF without MDD was 66.8 years compared to 64.4 years in the MDD group. More females were observed in the MDD cohort (51% vs 36.4%). The most common primary expected payer was Medicare/Medicaid in both groups (81.5% vs 79.6%). MDD patients were admitted to teaching hospitals more than the non-MDD group (72.2% vs 64.9%). Non-MDD patients had a higher amount of subjects in the 0-25th percentile group than MDD patients (32.8% vs. 28.8%). More significant facilities and home healthcare discharges were noted in the depression group than the non-depression group (41.0% vs 36.2%). Patients with MDD had a more significant comorbidity burden with a mean Elixhauser Comorbidity Score (6.3 vs 4.8). The HFrEF with MDD group had higher comorbidity of obesity, OSA, hypertension, diabetes mellitus, prior stroke, end-stage renal disease (ESRD), anemia, hypothyroidism, and anxiety disorder compared to the non-MDD group.

**Table 2:** Baseline characteristics for heart failure patients with and without depression: stratified by ejection fraction, National Readmission Database, 2018 ADHD = attention-deficit/hyperactivity disorder; CAD = coronary artery disease; CKD = chronic kidney disease; ESRD = end-stage renal disease; HFpEF = heart failure preserved ejection fraction; HFrEF = heart failure reduced ejection fraction; PTSD = post-traumatic stress disorder; OSA = obstructive sleep apnea; no. = number ^a^Represents Medicare and Medicaid. ^b^Small bed-size hospitals contain 1-250 beds, medium bed-size contains 50-450 beds and large bed-size hospitals contain 450+ beds. ^c^A hospital is considered a teaching hospital if it has one or more Accreditation Council for Graduate Medical Education (ACGME) approved residency program, is a member of the Council of Teaching Hospitals (COTH), or has a ratio of full-time equivalent interns and residents to beds of 0.25 or higher (https://www.hcup-us.ahrq.gov/db/vars/hosp_bedsize/nrdnote.jsp). ^d^Represents a quartile classification of the estimated median household income of residents in the patient’s ZIP Code. The quartiles are identified by values of one to four, indicating the poorest to wealthiest populations. Because these estimates are updated annually, the values of one to four vary by year (https://www.hcup-us.ahrq.gov/db/vars/zipinc_qrtl/nrdnote.jsp). ^e^Disposition to skilled nursing facility (SNF), intermediate care facility (ICF), Hospice – medical facility, long-term care hospital swing bed, rehabilitation facility, nursing facility certified by Medicaid or transfer to a psychiatric hospital (https://www.hcup-us.ahrq.gov/db/vars/dispuniform/nrdnote.jsp). ^f^ICD-10 codes were utilized to identify respective comorbidity as per Table 1.

Variables	HFrEF			HFpEF		
	Depression (N = 6642)	No depression (N = 59,761)	p-Value	Depression (N = 4643)	No depression (N = 31,951)	p-Value
Age (years) (mean)	64.4	66.8	<0.01	71.2	74.5	<0.01
18-49	18.6 (%)	15.5 (%)		7.3 (%)	5.6 (%)	
50-64	28.3 (%)	27.1 (%)		21.9 (%)	17.3 (%)	
65-74	22.7 (%)	20.3 (%)		25.2 (%)	19.7 (%)	
>75	30.4 (%)	37.0 (%)		45.5 (%)	57.3 (%)	
Indicator of gender			<0.01			<0.01
Male	48.7 (%)	63.6 (%)		30.2 (%)	41.0 (%)	
Female	51.2 (%)	36.4 (%)		69.8 (%)	58.9 (%)	
Admission day			0.33			0.68
Weekday	76.9 (%)	76.0 (%)		75.7 (%)	76.0 (%)	
Weekend	23.1 (%)	24.0 (%)		24.3 (%)	23.9 (%)	
Primary insurance			<0.01			0.22
Federal/state^a^	81.5 (%)	79.6 (%)		89.3 (%)	88.2 (%)	
Private	16.2 (%)	15.6 (%)		9.5 (%)	10.3 (%)	
Uninsured	2.2 (%)	4.7 (%)		1.1 (%)	1.4 (%)	
Hospital bed size^b^			0.06			0.11
Small/Medium	44.1 (%)	46.6 (%)		50.7 (%)	53.0 (%)	
Large	55.9 (%)	53.4 (%)		49.2 (%)	46.9 (%)	
Hospital teaching status^c^			<0.01			0.03
Non-teaching	27.8 (%)	35.1 (%)		37.2 (%)	40.2 (%)	
Teaching	72.2 (%)	64.9 (%)		62.8 (%)	59.8 (%)	
Median household income by zip-code, percentile^d^			<0.01			0.03
0-25th	28.8 (%)	32.8 (%)		25.2 (%)	26.3 (%)	
26-50th	29.1 (%)	29.7 (%)		29.7 (%)	32.2 (%)	
51-75th	24.7 (%)	22.8 (%)		26.4 (%)	24.3 (%)	
76-100th	17.4 (%)	14.7 (%)		18.5 (%)	17.1 (%)	
Disposition			<0.01			<0.01
Home/self-care	55.3 (%)	59.2 (%)		42.5 (%)	46.8 (%)	
Short-term hospital	1.6 (%)	1.9 (%)		1.1 (%)	1.1 (%)	
Facility^e^	16.9 (%)	14.1 (%)		26.5 (%)	23.5 (%)	
Home health care	24.1 (%)	22.1 (%)		28.7 (%)	27.4 (%)	
Against medical advice	1.9 (%)	2.6 (%)		1.0 (%)	1.0 (%)	
Elixhauser Comorbidity Index Score			<0.01			<0.01
1	0 (%)	2.1		0	0.9	
2	1.1 (%)	7.8		0.7	5.1	
3	5.4 (%)	15.4		3.4	12.3	
>4	93.4 (%)	74.6		95.8	81.5	
Mean Elixhauser Comorbidity Score	6.3	4.8	<0.01	6.4	5.2	<0.01
Comorbidities^f^						
Atrial fibrillation	37.3 (%)	37.6 (%)	0.74	43.9 (%)	48.6 (%)	<0.01
Obesity	24.3 (%)	17.1 (%)	<0.01	35.8 (%)	26.2 (%)	<0.01
OSA	19.2 (%)	10.5 (%)	<0.01	24.2 (%)	16.3 (%)	<0.01
Hypertension	34.0 (%)	31.3 (%)	0.01	38.6 (%)	38.1 (%)	0.67
Diabetes mellitus	35.2 (%)	31.5 (%)	<0.01	38.5 (%)	34.2 (%)	<0.01
CAD	51.3 (%)	51.5 (%)	0.85	35.8 (%)	37.4 (%)	0.17
Prior stroke	11.2 (%)	8.5 (%)	<0.01	11.9 (%)	9.9 (%)	<0.01
CKD stage >3	25.9 (%)	24.6 (%)	0.16	24.7 (%)	26.2 (%)	0.15
ESRD	2.7 (%)	2.0 (%)	0.02	2.1 (%)	2.6 (%)	0.22
Peripheral vascular disease	19.9 (%)	19.0 (%)	0.23	14.8 (%)	13.1 (%)	0.03
Anemia	28.8 (%)	24.3 (%)	<0.01	33.8 (%)	31.3 (%)	0.01
Hypothyroidism	19.5 (%)	13.2 (%)	<0.01	24.5 (%)	19.9 (%)	<0.01
Alcohol	6.5 (%)	6.8 (%)	0.51	3.4 (%)	2.8 (%)	0.18
Smoking	50.3 (%)	46.2 (%)	<0.01	45.1 (%)	39.3 (%)	<0.01
Cocaine use	2.4 (%)	2.4 (%)	0.82	0.6 (%)	0.5 (%)	0.60
Schizophrenia	1.3 (%)	1.0 (%)	0.09	1.0 (%)	0.8 (%)	0.27
Bipolar disorder	0.3 (%)	1.5 (%)	<0.01	0.4 (%)	1.7 (%)	<0.01
Anxiety disorder	34.9 (%)	8.4 (%)	<0.01	35.3 (%)	9.2 (%)	<0.01
PTSD/adjustment disorder	3.4 (%)	0.9 (%)	<0.01	2.2 (%)	0.7 (%)	<0.01
ADHD	1.9 (%)	0.3 (%)	<0.01	0.4 (%)	0.1 (%)	<0.01

In the HFpEF cohort, patients without MDD were older, with a mean age of 74.5 years compared to 71.2 years. Female patients were more prevalent in the MDD group than without depression (69.8% vs 58.9%). HFrEF with and without MDD had non-significant differences in day of admission, primary insurance, and hospital bed size. Facility discharge and discharges with home healthcare were higher in the MDD group (55.2% vs 50.9%: p <0.01). HFpEF patients with MDD had a more significant comorbidity burden with a mean Elixhauser comorbidity score (6.4 vs 5.2: p <0.01). Hospitalizations with MDD had higher comorbidity of obesity, OSA, diabetes mellitus, hypothyroidism, smoking, and anxiety.

Primary outcome: composite of all-cause readmissions and mortality in 30 days

In the HFrEF cohort, a total of 6642 indexes HFrEF with MDD hospitalizations were discharged alive. The composite outcome of all-cause readmission and mortality at 30 days occurred in 20.1% HFrEF patients with MDD compared to 20.0% in patients without MDD. Adjusted OR was 0.94 (95% CI: 0.85-1.04) (Table 3).

**Table 3:** Summary of outcomes of the study including rate and odds ratio of HFrEF and HFpEF with and without major depressive disorder HFpEF = heart failure with preserved ejection fraction; HFrEF = heart failure with reduced ejection fraction ^a^Odds ratio for heart failure with depression compared to heart failure without depression. ^b^Data were adjusted for age, gender, hospital bed size, hospital teaching status, median household income, disposition, Elixhauser comorbidity index, atrial fibrillation, obesity, obstructive sleep apnea, hypertension, diabetes mellitus, coronary artery disease, prior stroke, chronic kidney disease, anemia, hypothyroidism, smoker, schizophrenia, bipolar disorder, anxiety disorder, post-traumatic stress disorder/adjustment disorder, and attention-deficit/hyperactivity disorder. ^c^Primary outcome consisted of combined 30-day all-cause readmission and mortality.

Heart failure with reduced ejection fraction						
Outcome	Depression (n = 6642)	No depression (n =59,761)	Unadjusted odds ratio^ a^	p-Value	Adjusted odd ratio^a,b^	p-Value
Primary outcome^c^	1335 (20.1%)	11996 (20.0%)	1.00 (0.92-1.09)	0.97	0.94 (0.85-1.04)	0.27
30 day all-cause readmission	1146 (17%)	9759 (16.3%)	1.07 (0.97-1.17)	0.16	0.94 (0.85-1.05)	0.27
30-day mortality	241 (3.6%)	2778 (4.8%)	0.75 (0.60-0.94)	0.01	0.79 (0.62-1.00)	0.05
Heart failure with preserved ejection fraction						
Outcome	Depression (n = 4643)	No depression (n =31,951)	Unadjusted odds ratio^a^	p-Value	Adjusted odd ratio^a,b^	p-Value
Primary outcome^c^	861 (18.5%)	5928 (18.5%)	1.00 (0.88-1.12)	0.99	0.93 (0.81-1.07)	0.32
30-day all-cause readmission	774 (16.6%)	4942 (15.4%)	1.09 (0.96-1.23)	0.15	0.93 (0.81-1.07)	0.32
30-day mortality	87 (1.9%)	985 (3.0%)	0.60 (0.44-0.82)	<0.01	0.69 (0.50-0.95)	0.02

For the HFpEF cohort, a total of 4643 indexes HFpEF with MDD hospitalizations were discharged alive. The composite outcome of all-cause readmission and mortality at 30 days occurred in 18.5% on both HFpEF with and without MDD. Adjusted odds ratio (OR) was 0.93 (95% CI: 0.81-1.07). Lower median household income, leaving against medical advice, and higher Elixhauser Comorbidity Index Score were identified as major predictors of readmission and mortality in HFrEF and HFpEF groups (Table 4).

**Table 4:** Unadjusted predictors of combined 30-day all-cause readmission and mortality for each baseline characteristics ADHD = attention-deficit/hyperactivity disorder; CAD = coronary artery disease; CKD = chronic kidney disease; ESRD = end-stage renal disease; HFpEF = heart failure preserved ejection fraction; HFrEF = heart failure reduced ejection fraction; PTSD = post-traumatic stress disorder; OSA = obstructive sleep apnea

Variables	HFrEF		HFpEF	
	Unadjusted odds ratio	p-Value	Unadjusted odds ratio	p-Value
Age	1.0	0.56	0.99	0.32
Gender				
Male	Reference		Reference	
Female	0.9	<0.01	1.00	0.81
Admission day	1.05	0.11	1.08	0.06
Primary insurance				
Federal/state	Reference		Reference	
Private	0.68	< 0.01	0.77	< 0.01
Uninsured	0.76	< 0.01	0.94	0.75
Hospital bed size				
Small/medium	Reference		Reference	
Large	1.00	0.80	1.12	< 0.01
Hospital teaching status				
Non-teaching	Reference		Reference	
Teaching	0.93	0.05	0.94	0.22
Median household income category for patient’s zip-code				
0-25th	Reference		Reference	
26-50th	0.83	< 0.01	0.92	0.11
51-75th	0.85	< 0.01	0.89	0.04
76-100th	0.78	< 0.01	0.80	< 0.01
Disposition				
Home	Reference		Reference	
Short-term hospital	1.19	0.11	1.84	< 0.01
Facility	1.04	0.38	1.15	< 0.01
Home health care	1.11	< 0.01	1.19	< 0.01
Against medical advice	2.76	< 0.01	1.94	< 0.01
Elixhauser Comorbidity Index Score				
1	Reference		Reference	
2	0.85	0.27	1.16	0.58
3	1.08	0.56	1.31	0.30
>4	1.45	< 0.01	1.99	< 0.01
Co-morbidities				
Atrial fibrillation	1.08	< 0.01	1.18	< 0.01
Obesity	0.88	< 0.01	0.92	0.06
OSA	0.91	0.05	1.00	0.95
Hypertension	1.10	< 0.01	1.07	0.09
Diabetes mellitus	1.14	< 0.01	1.13	< 0.01
CAD	1.18	< 0.01	1.18	< 0.01
Prior stroke	1.16	< 0.01	1.14	0.04
CKD stage >3	1.36	< 0.01	1.40	< 0.01
ESRD	1.81	< 0.01	1.54	< 0.01
Peripheral vascular disease	1.20	< 0.01	1.13	0.02
Anemia	1.31	< 0.01	1.24	< 0.01
Hypothyroidism	1.13	< 0.01	1.09	0.05
Alcohol	0.92	0.18	0.95	0.68
Smoking	0.91	< 0.01	0.97	0.42
Cocaine use	1.30	< 0.01	1.49	0.08
Schizophrenia	1.55	< 0.01	0.86	0.54
Bipolar disorder	1.06	0.58	1.21	0.16
Anxiety disorder	1.15	<0.01	1.05	0.40
PTSD/adjustment disorder	1.10	0.42	0.77	0.20
ADHD	0.74	0.21	1.03	0.94

Secondary outcomes

All-Cause Readmission Rate at 30 Days

In the HFrEF cohort, 17% of patients with MDD were readmitted within 30 days. The three most common principal diagnoses for readmissions in the MDD cohort were acute heart failure (44.9%), sepsis (4.5%), and acute kidney failure (2.9%). HFrEF without MDD had a 16.3% rate of readmission. Comparing HFrEF patients with MDD and without MDD, the adjusted OR was 0.94 (95% CI: 0.85-1.05) (Table 3).

In the HFpEF cohort, the 30-day all-cause readmission rate for patients with MDD was 16.6% compared to 15.4% in the non-MDD group. The three most common principal diagnoses for readmissions in HFpEF with MDD were acute heart failure (41.0%), sepsis (7.2%), and acute and chronic hypoxic respiratory failure (4.9%). Adjusted OR was 0.93 (95% CI: 0.81 - 1.07) when compared HFpEF with MDD to without MDD.

All-Cause Mortality Rate at 30 Days

 In the HFrEF cohort, the 30-day mortality rate in the MDD and non-MDD groups was 2.8% and 3.6%, respectively. MDD was associated with lower mortality than non-MDD in HFrEF with an adjusted OR of 0.79 (95% CI: 0.62-1.00). In the HFpEF cohort, the 30-day mortality rate in the MDD group was 1.9% and 3% in the non-MDD group. HFpEF patients with MDD had lower mortality than those without MDD, with an adjusted OR of 0.69 (95% CI: 0.5-0.95).

Resource utilization

In the HFrEF cohort, patients with MDD had a longer mean length of stay (6.7 vs 5.7 days; p <0.01) (Table 5). HFrEF with MDD had higher mean total initial hospitalization charges compared to those without MDD (USD $69,810 vs $62,935; p = 0.03). Readmission cost was higher in both HFrEF with and without MDD compared to the index admission.

**Table 5:** Resource utilization HFpEF = heart failure preserved ejection fraction; HFrEF = heart failure reduced ejection fraction; LOS = length of hospital stays; SD = standard deviation

Resource outcomes	HFrEF			HFpEF		
	Depression	No depression	p-Value	Depression	No depression	p-Value
Mean total charges ($)						
Index (initial admission)	69,810	62,935	0.03	44,512	42,165	0.13
Readmission	74,591	66,230	0.10	58,794	59,529	0.87
Mean LOS (±SD) (days)	6.6 (±8.9)	5.7 (±7.6)	<0.01	5.6 (±5.5)	5.3 (±5.5)	<0.01

In the HFpEF cohort, patients with MDD had a longer length of stay than the non-MDD group (5.6 vs 5.3 days; p <0.01). The mean total hospitalization charges were not statistically different between MDD and non-MDD groups ($44,512 vs $42,165; p = 0.13).

## Discussion

This is the first study investigating the association of MDD and HF in terms of HFrEF and HFpEF. Using NRD 2018, a large US national database, we found a total prevalence of 11% for all types of HF patients with MDD. Patients with HFrEF had less prevalence of MDD (10%) compared to HFpEF (13.9%). The combined rate of all-cause readmission and mortality at 30 days was not statistically different in both types of HF patients (HFrEF and HFpEF) with or without MDD. Both types of heart failure with concomitant depression were not associated with all-cause 30-day readmissions but were associated with decreased mortality rates in both groups.

Heart failure patients with MDD were younger and more likely to be female than the group without MDD. Patients in the heart failure with preserved ejection fraction group were older than HFrEF with an average age of 72-75 years compared to 64-67 years. HFrEF with MDD had more comorbidity of obesity, OSA, hypothyroidism, tobacco use, anemia, and a higher Elixhauser Comorbidity Index Score than those without MDD. This pattern was similar to HFpEF with MDD, except that the HFpEF without MDD group had a higher prevalence of atrial fibrillation. These comorbidities were associated with increased HF mortality and rehospitalization, except for obesity [17-20]. Obesity is highly prevalent in HF groups and interestingly associated with lower mortality in HF patients [18]. In our study, obesity was more commonly associated with the MDD group, which could have contributed to lesser mortality in the MDD group for both types of HF. In our study, underlying diseases involving psychiatric issues, such as anxiety disorder and PTSD, were more prevalent in HF with MDD than patients without MDD. Lin et al., had shown that anxiety was not associated with readmission or mortality at an 18-month duration in the HFrEF group but had increased readmission and mortality in the HFpEF group [21]. Similarly, in our study, anxiety disorder was more prevalent in MDD groups and did not result in a higher readmission rate. However, we noticed lower mortality in MDD groups. This difference could be related to the shorter 30-day follow-up period in our study. Patients with HFrEF with MDD were less likely to be discharged to home compared to those without MDD. On the contrary, HFpEF patients with MDD were likely to discharge home compared to those without MDD. These comorbidities affected outcomes differently across all HF patients, both with and without MDD. Therefore, we used multivariate regression analysis to adjust for all the comorbidities to find the actual impact of MDD on HF.

We found that MDD was not associated with an all-cause readmission rate for both types of HF. This finding was different from a previous NRD study that used the 2010-2014 database which showed an increased readmission rate in the MDD group compared to non-MDD [2]. This difference could be related to the study's inclusion of all types of heart failure but not specific HFrEF and HFpEF ICD codes. The current expert recommendations for depression in HF patients are similar to non-HF patients however, antidepressant efficacy has not been demonstrated yet in the HF patients but the more effective therapies are psychotherapy and care management [22]. This is unfortunately not measurable in our study due to the inherent limitation of the database itself. In our study, HFrEF and HFpEF with MDD were associated with similar readmission rates and significantly lower mortality at 30 days compared to those without MDD. This finding is different from the previous studies that showed increased mortality in HF patients with depression of two to four-fold [1,2]. In theory, HF patients with MDD could have received more attention, follow-ups, and multidisciplinary care compared to HF patients without MDD. This could contribute to reduced mortality. Preliminary results of the Blended Collaborative Care for Heart Failure and Co-morbid depression trial (NCT02044211) in HFrEF patients with depression showed that collaborative care of psychiatrists, cardiologists, internists, and nurses, reduced patients mood symptoms but not mortality compared to HF patients without depression [23]. Unfortunately, NRD does not contain the medications, psychotherapy, and social support data that could influence a positive outcome. More research is needed to pinpoint the most effective treatment for HF patients with depression.

Future perspective

Prospective studies are needed to strengthen the association and establish causality for decreased mortality with patients with MDD compared to HF patients without MDD. Better screening, treatment, and management of depression could reduce the worsening outcomes in HF patients. Studies with more extended follow-up periods are needed to assess long-term readmission differences in this patient population. 

Limitations

We acknowledge several limitations in this study. First, NRD relies on administrative coding, and data depends on the rigor of the institutions involved. Second, NRD does not contain information on medications, laboratory values, echocardiographic data, and other treatment modalities like cognitive behavioral therapy, limiting our assessment of the studied association. Third, the database does not account for deaths outside the hospital, impacting the mortality association. Despite these limitations, this large population study contains half of the US rehospitalized patient population and may provide significant external validity and generalizability findings.

## Conclusions

The prevalence of MDD in HFpEF was higher than HFrEF at 13.9% and 10%, respectively. Concomitant MDD in HF patients was not associated with the combined outcome of in-hospital mortality and rehospitalization at 30 days. However, the secondary outcome of 30-day mortality was significantly lower in HFrEF and HFpEF patients with MDD compared to patients without MDD. There are several inherent limitations of the NRD database which limits us from examining the factors contributing to the decreased mortality in the MDD group. Further studies are needed with a longer follow-up period and increased granularity of the data to verify the results of this study and also to determine the treatment that influences readmission and mortality in HF patients with MDD.
